# Detection and Isolation of Viable Mouse IL-17-Secreting T Cells

**DOI:** 10.3791/1037

**Published:** 2008-12-18

**Authors:** Anna Foerster, Mario Assenmacher, Michaela Niemoeller, Elly Rankin, Mariette Mohaupt, Anne Richter

**Affiliations:** Miltenyi Biotec,GmbH

## Abstract

The MACS Cytokine Secretion Assay technology allows detection of secreted cytokines on the single cell level and sensitive isolation of viable cytokine-secreting cells. In order to label IL-17-secreting cells, a single cell suspension of mouse splenocytes is prepared and stimulated at 37°C with PMA/ionomycin to induce cytokine secretion. To stop secretion cells are then placed on ice and are exposed to the IL-17 Catch Reagent   a bi-specific antibody that binds to CD45 on the cell surface of leukocytes and to IL-17 as it is secreted and caught near the cell surface. Secretion is then re-started by increasing the temperature to 37°C and IL-17 is trapped by the Catch Reagent. Secretion is then stopped again, by placing cells on ice. To detect the trapped IL-17, cells are incubated with a second IL-17-specific antibody conjugated to biotin and an Anti-Biotin-PE antibody. Cells can now be directly analyzed by flow cytometry or prepared for isolation and enrichment by subsequent labeling with Anti-PE conjugated MicroBeads.

**Figure:**
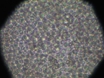


## Protocol

### Preparing the Reagents

Make  a buffer containing PBS with BSA (0.5%), and 2 mM EDTA. Because air bubbles can block MACS separation columns, the buffer needs to be degassed and stored at 2-8°C before use.
We use RPMI 1640 medium containing 5% mouse serum. The culture medium should not contain BSA or FCS, as these compounds will alter the specificity of cell stimulation.
The Mouse IL-17 Secretion Assay - Cell Enrichment and Detection Kit from Miltenyi-Biotec. The kit contains the following components: the IL-17 Catch Reagent, the IL-17 Detection Antibody (Biotin), the Anti-Biotin-PE, and the Anti-PE MicroBeads.

### Stimulating the Splenocytes

This protocol is performed in the presence of a negative and positive control, such as unstimulated splenocytes and a counterstain for T cells.  This protocol is carried out using sterile technique.
Prepare a single cell suspension of mouse splenocytes that were isolated using the gentleMACS™ Dissociator. The concentration of cells should be predetermined via cell counting.  Pellet the cells at 200×g for 10 minutes at room temperature.
Following centrifugation, aspirate the supernatant off the pellet using a pipette.  Do not decant the tube to avoid loss of the pellet.
Now, resuspend the cells in the culture medium and then add to a well. Add sufficient medium for a concentration of ten million cells per mL and five million cells per square cm.
To stimulate an immune response in our resuspended cells, we add ionomycin (1 μg/mL) and PMA (10 ng/mL) to the sample and mix the solution by gently pipetting up and down. Then the wells are labeled accordingly.
Now, we will incubate our cells for 3 hours at 37 °C with no mixing to start the stimulation period. Proceed to IL-17 analysis 3 hours from the onset of stimulation, so plan accordingly.
To stop secretion, cells are placed on ice and we collect the stimulated cells by gently pipetting up and down with cold buffer. Cells are then transferred from the well to a tube and are washed a second time.
To ensure that all cells are collected, it’s a good idea to check your dish under a microscope. If cells still remain attached, you can collect the remaining cells by rinsing the dish with cold buffer.  Any cell clumps in your cell suspension can be removed using the pre-separation filters.

### Labeling the Cells with Catch Reagent

It is paramount to note, that this assay works optimally if less than 2% of IL-17- secreting cells are present.
If the concentration of IL-17 secreting cells is expected to be greater than 2% adjust  volumes accordingly.
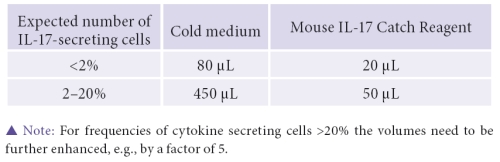
One potential pitfall of this procedure is cross contamination of the Catch Reagent, which can occur during labeling when the bi-specific antibody binds to a non-secreting T cell and traps IL-17 secreted from a neighboring lymphocyte, thereby generating false positives.
In order to circumvent this problem, it is critical to cool cells down prior to labeling and work with cold buffer to slow diffusion of IL-17  and avoid this cross contamination. In addition to keeping the cells cold, they must be kept at a defined concentration.
To begin the labeling procedure, we use ten million cells in a 15 ml closable tube. If higher cell numbers have to be used, simply scale up all volumes accordingly. Once the optimal cell concentration has been obtained, wash the cells by adding 10 ml of cold buffer.
Spin down the cells at 300×g for 10 minutes in a refrigerated centrifuge (2–8 °C).
Following centrifugation, aspirate the supernatant completely using a pipette. Do not decant the supernatant as this will lead to cell loss and imprecise volumes. Repeat the washing step which consists of adding 10ml of cold buffer, centrifugation, and aspiration.
Now that we have a pellet of desired purity, resuspend the cells in 80 μL of cold culture medium. To label them, we will now add 20 μL of Mouse IL-17 Catch Reagent.
Incubate the cells for 5 minutes on ice.
After the 5 minute incubation period on ice, remove the tube and dilute the cells in 10 ml of 37°C warm medium. Then secure the tube on the MACSmix Tube Rotator and incubate the tube at 37°C for 45 min under continuous motion. Increasing the temperature to 37°C will re-start cytokine secretion.

### Labeling the Cells with IL-17 Detection Antibody (Biotin) and Anti-Biotin-PE

After the 45 minute secretion period at 37°C, place the tube immediately on ice. This will stop cytokine secretion. From here on it is critical to keep cells on ice. Working with cold buffer will avoid cross contamination of the Catch Reagent.
Fill up the tube with cold buffer and centrifuge at 300xg at 2–8 °C for 10 min. Aspirate the supernatant completely. Repeat the wash step one more time.
The cell pellet is resuspended in 80 μL of cold buffer and the tube is placed on ice.
To avoid unspecific binding of the antibody, we recommend the addition of 10µl FcR Blocking Reagent and incubate on ice for 5 min. Then we add 20 μL of Mouse IL-17 Detection Antibody (Biotin) that is conjugated to biotin and incubate for 10 minutes on ice.
Once again, wash cells as we have done before by adding 10 mL of cold buffer and centrifuge at 300xg at 2–8 °C for 10 min.
Now aspirate the supernatant and resuspend the cell pellet in 80 µl of cold buffer.
Add 20 μL of our secondary antibody, Anti-Biotin-PE.
Mix the cells and secondary antibody solution, and again incubate the tube for 10 minutes on ice.
Once this second incubation is complete, wash the cells with 10 mL cold buffer and centrifuge.
The pellet can now be resuspended in 500 μL of cold buffer.
If the cells have to be separated for further downstream analysis, they can be magnetically labeled with Anti-PE-MicroBeads and separated manually or automated using MACS Columns and MACS Separators.
We have now completed the labeling of the splenocytes and are ready for analysis.

### FACS Analysis

For our analysis, we will compare the stimulated and unstimulated splenocytes by flow cytometry.
Before flow cytometric analysis, cells are stained with propidium iodide at 0.5 μg/mL to gate out dead cells.  The MACSQuant™ Analyzer was loaded with 200,000 cells for each sample and we now are going to look at the scatter plots of relative antibody reactivity in the samples. Two important considerations for analysis of rare cells - like IL-17 positive  cells -  by flow cytometry are:
  setting a gate on lymphocytes in the forward scatter versus side scatter plot
  and gating out dead cells (stained with propidium iodide) and B-cells (which may cause unspecific background staining) to further enhance the sensitivity of detection
We used CD4-APC antibody to detect T cells and the CD45R/B220-PerCP antibody to detect the B cells. We see the forward and side scatter properties of the samples and apply a gate on the lymphocyte population.
A second gate was applied to see the stained dead cells and stained B cells (Y axis). These cells are excluded from the T cell analysis.
In this example, which is essentially our negative control, we can see that there are very few IL-17 secreting CD4 positive T cells in unstimulated samples – approximately 0.003% (Figure 4).  Looking at the stimulated T cell population, we can see a substantial number of CD4 positive T cells that secrete IL-17 – about 0.367% (Figure 5A) before separation and 60.76% after magnetic separation with MACS Technology (Figure 5B).

## Disclosures

<p>All protocols and data sheets are available at www.miltenyibiotec.com. </p><p> Warnings<br /> Reagents contain sodium azide. Under acidic conditions sodium azide yields hydrazoic acid, which is extremely toxic. Azide compounds should be diluted with running water before discarding. These precautions are recommended to avoid deposits in plumbing where explosive conditions may develop.

## Discussion

TH17 cells play an integral role in adaptive immunity and the inflammatory response and are a key component in driving autoimmune inflammation.

This video documents how to use Miltenyi’s Mouse IL-17 Secretion Assay – Cell Enrichment and Detection Kit (PE). When doing this procedure it’s important to have an estimation of the frequency of IL-17-secreting cells in your starting cell preparation. The appropriate cell concentration during the labeling and cytokine secretion period prevents cross-contamination by other cells in your suspension and assures reliable results.

